# New Appliances and Things Medical

**Published:** 1905-03-25

**Authors:** 


### NEW APPLIANCES AND THINGS MEDICAL.

[We shall be glad to receive, at our Office, 28 & 29 Southampton Street, Strand, London, W.C., from the manufacturers, specimens of all new preparations and appliances which may be brought out from time to time.]

### DYMAL.

(Messrs. Zimmer and Co, Frankfurt)

"Dymal" is an odourless, nearly tasteless powder, of a slightly pink colour. It occurs as a bye-product in the manufacture of mantles for incandescent gas-lights, and, chemically, is salicylate of didymium, having a formula $Di_2 (C_6 H_4 OH. COO)_6$ As a dusting power dymal is excellent on account of its antiseptic and siccative powers. It also makes a suitable and efficient ointment for most infective cutaneous eruptions, such as impetigo, etc, for which purpose it may be used in strengths of 5 per cent. or 10 per cent. There is probably a wide field of therapeutic usefulness for this new preparation.

